# Human anisakiasis in Italy: a retrospective epidemiological study over two decades

**DOI:** 10.1051/parasite/2018034

**Published:** 2018-07-30

**Authors:** Lisa Guardone, Andrea Armani, Daniele Nucera, Francesco Costanzo, Simonetta Mattiucci, Fabrizio Bruschi

**Affiliations:** 1 FishLab, Department of Veterinary Sciences, University of Pisa Via delle Piagge 2 56124 Pisa Italy; 2 Department of Agriculture, Forestry and Food Science, University of Turin Largo Braccini 2 10095 Grugliasco – Torino Italy; 3 Department of Public Health and Infectious Diseases, Section of Parasitology, “Sapienza – University of Rome”, Laboratory affiliated to Istituto Pasteur Italia-Fondazione Cenci Bolognetti P.le Aldo Moro, 5 00185 Rome Italy; 4 Department of Translational Research, N.T.M.S., School of Medicine, University of Pisa via Roma, 55 56126 Pisa Italy

**Keywords:** *Anisakis* sp., *Anisakis pegreffii*, zoonosis, diagnosis, anchovies, seafood safety

## Abstract

A retrospective analysis on human anisakiasis in Italy since its first description in 1996 was performed by conducting a literature search. Inclusion criteria based on the presence of a larva and on parasite identification were applied. Epidemiological data and clinical features were analysed. Particular attention was paid to the source of infection. In total, 73 cases were included in the analysis, while 34 were excluded. Cases were reported from eight Italian regions, most frequently Abruzzo, Apulia and Latium. The parasite was detected by endoscopy (51.4%) or laparotomy (48.6%). The site of infection was intestinal (42.5%), gastric (43.8%), oesophageal (1.4%) or ectopic (12.3%). Most of the parasites (71.0%) were identified as *Anisakis* sp. or *A. simplex* (*s.l.*). However, when molecular methods were used (21 cases), *A. pegreffii* was always identified. In most of the patients (65.7%), the source of infection was raw or undercooked anchovies, followed by “anchovies or sardines” (15.1%), generic “raw seafood” (15.1%), and sardines (1.4%). In only 2 cases (2.7%), the source was not available. This is the first systematic analysis of Italian cases of anisakiasis. The main conclusions derived from the results are: i) attention should be given to the history, in particular when raw marinated anchovies, proven to be the main source of human anisakiasis in Italy, are consumed; ii) in order to assess correct epidemiological data, a confirmed and specific etiological identification should always be sought.

## Abstract (fr)

Une analyse rétrospective sur l’anisakiase humaine en Italie depuis sa première description en 1996 a été réalisée en effectuant une recherche bibliographique. Des critères d’inclusion basés sur la présence d’une larve et sur l’identification des parasites ont été appliqués. Les données épidémiologiques et les caractéristiques cliniques ont été analysées. Une attention particulière a été accordée à la source de l’infection. Soixante-treize cas ont été inclus dans l’analyse, tandis que 34 ont été exclus. Les cas ont été signalés dans huit régions italiennes, le plus souvent en Abruzzes, Pouilles et Latium. Le parasite a été détecté par endoscopie (51.4 %) ou laparotomie (48.6 %). Le site d’infection était intestinal (42.5 %), gastrique (43.8 %), œsophagien (1.4 %) ou ectopique (12.3 %). La plupart des parasites (71.0 %) ont été identifiés comme *Anisakis* sp. ou *A. simplex* (*s.l.*). Cependant, lorsque des méthodes moléculaires ont été utilisées (21 cas), *A. pegreffii* a toujours été identifié. Chez la plupart des patients (65.7 %), la source d’infection était les anchois crus ou insuffisamment cuits, suivis par « anchois ou sardines » (15.1 %), « fruits de mer crus » (15.1 %) et sardines (1.4 %). Dans 2 cas (2.7 %) la source n’était pas disponible. Cette étude est la première analyse systématique des cas italiens d’anisakiase. Les principales conclusions tirées des résultats sont les suivantes: i) une attention particulière doit être portée à l’anamnèse, surtout lorsque les anchois marinés crus, qui sont la principale source d’anisakiase humaine en Italie, sont consommés; ii) une identification parasitologique doit toujours être effectuée pour valider les données épidémiologiques.

## Introduction

Parasitic zoonoses of food safety concern are generally underestimated but are increasing dramatically [20]. Since notification to public health authorities is not mandatory for most parasitic diseases, official reports do not reflect the true prevalence or incidence of these diseases [44]. In addition, while the importance of meat-borne parasitic zoonoses such as trichinellosis and cysticercosis has long been recognised, fish-borne parasitic diseases like opisthorchiasis, clonorchiasis, intestinal trematodiasis, diphyllobothriasis or anisakidosis have received, until two decades ago, less attention, despite the large numbers of human infections [30, 46, 85]. This is probably due to the fact that in the past, the risk of human infection with such parasites was considered to be limited to populations living in low- and middle-income countries in distinct geographic regions. However, the geographical limits and populations at risk have expanded because of growing international markets, improved transportation systems, demographic changes, an increasing population of susceptible persons, and changing culinary habits, leading to a renewed interest in these parasitic infections [41, 85].

“Anisakidosis” is the zoonotic disease caused by the third larval stage of anisakid nematodes, most frequently belonging to the *Anisakis* and *Pseudoterranova* genera, or, very rarely, to species of the genus *Contracaecum* [57]. Infection due to larvae of the genus *Anisakis* may be indicated as “anisakiosis” [57], even though the term “anisakiasis” is more frequently used [71, 110, 114]. Adult worms are found in the stomach of marine mammals (cetaceans); eggs are passed in their faeces and become embryonated in sea water. Crustaceans (krill) become infected with larval stages. When crustaceans are preyed on by fish or squid, the third stage larvae infect their viscera encysting on organ surfaces and, eventually, in the musculature [60, 76]. Humans represent an accidental host of these parasites; the zoonotic infection is acquired through the consumption of raw or undercooked infected marine fish or squids [68, 73]. The larvae do not develop further in humans; however, they can penetrate the gastrointestinal mucosa or, more rarely, other organs, often with clinical consequences. In addition, these parasites may generate potentially allergic reactions, characterised by urticaria or angio-oedema [11, 37, 75].

The first zoonotic case attributed to *Anisakis* species was described in the Netherlands around 1960, and correlated to the consumption of raw herrings [118]. Recent data stem from a systematic review of the literature by Orphanet, with an estimated worldwide incidence of 0.32/100,000) [88]. Japan and other Far-Eastern countries account for the vast majority of cases, reflecting the traditional frequent consumption of raw fish in these regions, where anisakiasis is considered a relatively common public health issue [11, 76, 90]. It is estimated that more than 2500 cases occur annually in Japan. In Europe, the exact incidence is difficult to establish, but it seems to be lower than 20 cases per country per year [2]. No data on human cases of anisakidosis are reported in the most recent European Union summary report on trends and sources of zoonoses, zoonotic agents and food-borne outbreaks [42]. The lack of data on certain parasitic zoonoses which are known to occur in the European Union in the EFSA reports has already been pointed out by other authors, who highlighted that few parasitic infections are considered [41]. In fact, the only parasitic zoonoses currently included are trichinellosis, echinococcosis and toxoplasmosis [42]. A very recent study estimated the incidence of anisakidosis in France by analysing data from the parasitology laboratories of university hospitals. A total of 37 cases in the years 2010–2014 were found [119], showing a decrease in comparison with a similar study conducted between 1985 and 1987 [54]. As regards the United States of America, an incidence of 10 cases per year was reported [2]; however, the exact frequency of anisakidosis is unknown [17].

In Italy, the first confirmed human case was described in 1996 [113]. Following this first description, the number of reported human cases in Italy has steadily increased. However, the real occurrence of anisakiasis in the Italian population could be underestimated, since scientific data are scattered and hospital reports are generally not published. As a consequence, contrasting estimates of the number of cases can be found [34, 38, 51, 76].

The aim of the present study was to conduct a retrospective analysis on the human cases of anisakiasis described in Italy since 1996. The epidemiological data and clinical features of the reported cases were analysed. Particular attention was paid to the main sources of infection of humans by *Anisakis* spp., whenever known.

## Materials and methods

### Literature search and selection of cases

To review cases of *Anisakis* spp. infections in humans in Italy, literature data were identified on the PubMed and Google Scholar databases using the following keywords: (*Anisakidae* OR *Anisakis*) AND (human OR human cases) OR (anisakidosis OR anisakiasis) AND Italy. The search was conducted using the terms together or combined differently in order to retrieve the maximum number of records (Appendix A). Then, the reference list of the screened articles was also checked for eligible new records. Only papers published in English, French, Italian and Spanish were included. The search was concluded in January 2018. In addition, 15 cases, reported in a thesis [40] and confirmed by the supervisor Dr. Paolo Fazii, Santo Spirito Hospital, Pescara (personal communication) were included.

After deduplication (conducted by comparing the sex, age, geographical location, clinical and parasitological features of the different patients and excluding overlapping cases), cases were considered eligible (confirmed cases) and included in the study when the following conditions were met: i) an *Anisakis* spp. larva was detected in the patient (by endoscopic removal or on histological examination following surgical removal of biopsy tissue), ii) identification of the parasite was performed, at least to genus level, by microscope observation of morphological characters referring to an *Anisakis* spp. larva, or to species level by the use of different molecular tools. The excluded cases were considered as “suspected”.

### Data Extraction

For each study, the following data were extracted using predefined data fields in an Excel file: number of patients involved, age, sex, geographical origin, source of infection, parasite detection and identification method, site of infection and number of larvae found, symptoms, time between the ingestion of infected food and the onset of symptoms, treatment, outcome, occurrence of allergic reactions, and seropositivity.

### Statistical analysis

Associations among variables were investigated using the chi-square test. Results were considered significant when *p* < 0.05 and highly significant when *p* < 0.001. Investigated couples of associated variables were: i) site of infection of the larva and time of symptom onset; ii) site of infection of the larva and treatment; iii) symptoms and time of onset; iv) symptoms and treatment. All the variable categories were mutually exclusive and each case was unambiguously classified. A multivariate analysis was conducted assuming as dependent variable the method used for larval detection (endoscopy/laparotomy), and the site of infection of the larva and the time of symptom onset as independent variables.

## Results

### Literature search and selection of cases

A total of 47 literature records were selected and further screened. Out of these 47 records, 28 reported cases that matched the inclusion criteria (presence of a larva and identification to genus or species level) were included in the study, while a further 19 were excluded (Table 1). The 28 publications included in the study described 65 putative cases. However, only 58 of them met the criteria and were further selected for the analysis. With the addition of the 15 cases reported in [40] and confirmed by P. Fazii, a total of 73 cases were included in the analysis. Likewise, 34 “suspected” cases (27 from the 19 excluded records and 7 from the 28 included records) were excluded.

**Table 1.** List of included and excluded cases reported in scientific articles, with some of the most relevant data.

Reference	Number of patients	Site of infection	Method used for larval detection	Etiological agent
Included records				
Stallone et al. [113]	1	Gastric	*–*	*Anisakis* sp.
Cancrini et al. [24]	1	Extra-gastrointestinal	Laparotomy	*Anisakis* sp.
Cancrini et al. [23]	1	Gastric	Endoscopy	*Anisakis* sp.
D’Amelio et al. [35]	1	Gastric	Endoscopy	*A. pegreffii* *
Maggi et al. [64]	3 (4)^a^	Gastric (1), intestinal (1), extra-gastrointestinal (1)	Laparotomy	*A. simplex* (*s.l.*)
Pampiglione et al. [90]	11	Gastric (2), intestinal (3), extra-gastrointestinal (5), spleen (1)	Endoscopy, laparotomy	*Anisakis* sp.
Caramello et al. [25]	1	Intestinal	Laparotomy	*Anisakis* sp.
Moschella et al. [83]	1	Intestinal	Laparotomy	*Anisakis* sp.
De Nicola et. al. [39]	1	Intestinal	Laparotomy	*A. simplex*
Montalto et al. [82]	1	Intestinal	Laparotomy	*A. simplex* (*s.l.*)
Pellegrini et al. [91]	1	Intestinal	Laparotomy	*A. simplex (s.l.)*
Fazii et al. [47]	3	Gastric (2), intestinal (1)	Endoscopy	*Anisakis* sp.
Avellino et al. [12]	1	Esophagus	Endoscopy	*A. pegreffii* *
Ugenti et al. [117]	3	Gastric	Endoscopy	*A. simplex* (*s.l.*)
Fumarola et al. [49]	2	Gastric	Endoscopy	*A. pegreffii* *
Marzocca et al. [67]	1	Intestinal	Laparotomy	*A. simplex* (*s.l.*)
Aloia et al. [3]	1	Intestinal	Endoscopy	*A. simplex* (*s.l.*)
Mattiucci et al. [74]	1	Intestinal	Laparotomy	*A. pegreffii* *
Pontone et al. [96]	1(2)^b^	Gastric	Endoscopy	*Anisakis* sp.
Mattiucci et al. [71]	8	Gastric (6), (GAA) (2)	Endoscopy	*A. pegreffii* *
Mumoli and Merlo [84]	1	Intestinal	Endoscopy	*A. simplex* (*s.l.*)
Andrisani et al. [5]	1	Intestinal	Endoscopy	*Anisakis* sp.
Baron et al. [15]	1(6)^c^	Intestinal	Laparotomy	*Anisakis* sp.
Mariano et al. [65]	1	Gastric	Endoscopy	*Anisakis* sp.
Carbotta et al. [26]	1	Intestinal	Laparotomy	*Anisakis* sp.
Mattiucci et al. [70]	5	Gastric (GAA)	Endoscopy	*A. pegreffii* *
Mattiucci et al. [75]	3	Gastric (1), intestinal (2)	Endoscopy, laparotomy	*A. pegreffii* *
Palma et al. [89]	1	Gastric	Endoscopy	*Anisakis* sp.
Total (28 records)^e^	58 (65)^d^			
Excluded records				
Ioli et al. [55]	1	*–*	*–*	*–*
Riva et al. [101]	4	*–*	*–*	*–*
Bavastrelli et al. [16]	1	*–*	*–*	*–*
Testini et al. [116]	1	*–*	*–*	*–*
Piscaglia et al. [95]	1	*–*	*–*	*–*
Pezzilli et al. [93]	1	*–*	*–*	*–*
Biondi et al. [18]	1	*–*	*–*	*–*
Rea et al. [99]	1	*–*	*–*	*–*
Zullo et al. [122]	1	*–*	*–*	*–*
Filauro et al. [48]	1	*–*	*–*	*–*
Sola et al. [111]	1	*–*	*–*	*–*
Taranto et al. [115]	6	*–*	*–*	*–*
Fava et al. [45]	1	*–*	*–*	*–*
Griglio et al. [51]	1	*–*	*–*	*–*
Bucci et al. [21]	1	*–*	*–*	*–*
Cesaro et al. [29]	1	*–*	*–*	*–*
Marra et al. [66]	1	*–*	*–*	*–*
Zippi et al. [121]	1	*–*	*–*	*–*
Zanelli et al. [120]	1	*–*	*–*	*–*
Total (19 records)	27	*–*	*–*	*–*

a Four reported cases but only 3 confirmed by histology;

b In the second case no larva was found;

c Six cases are reported but only one shows photos and description of the histological section;

d Total number of cases reported in the 27 articles;

e In addition, 15 cases reported in a thesis (De Rosa [40]) and confirmed by Dr. Fazii, not shown in the table, were included in the analysis.

* Molecular identification.

### Age distribution and sex ratio

Age was available for 58 patients: the mean age was 44.4 (*SD* = 12.9) and the age range 19–86. The disease showed the highest frequency in the thirties age group (29.3%), followed by forties (25.9%), fifties (25.9%), over sixties (10.3%) and twenties (6.9%). Similarly, also in the study of Sohn et al. [110], the highest relative frequency of occurrence was found in the thirties and forties age groups and the lowest in teens. A higher frequency in adults than in children was also observed by Pampiglione et al. [90].

As regards the sex ratio, gender was available for 60 patients: a slight difference, although not significant, was observed as 25 were men and 35 were women. A significant predominance of women was found in France [119].

### Geographical distribution

**Figure 1.**
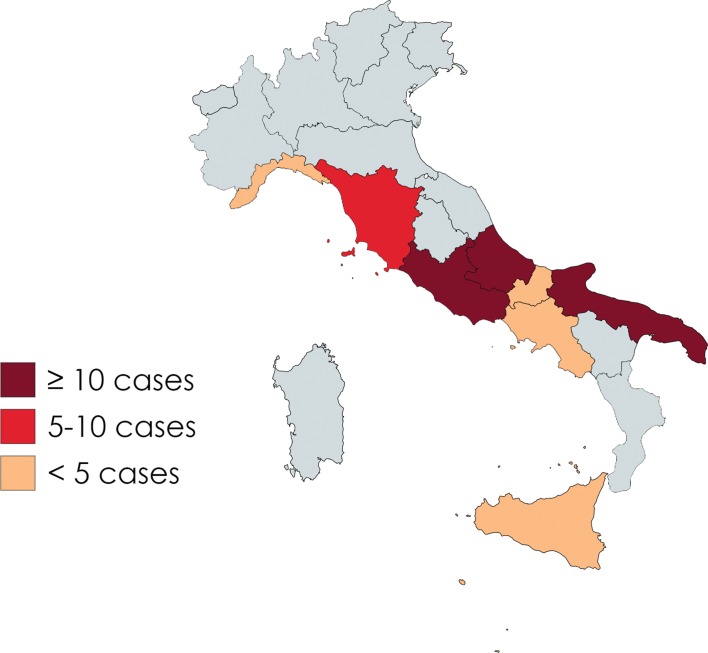
Map of the distribution of the Italian cases of anisakiasis included in the present study, divided per region. Created with Mapchart.net.

The cases for which a geographical origin was available (*N* = 67, 91.8%) were distributed in eight Italian regions. In 13 of these cases, the geographical origin was attributed on the basis of the hospital where the patient was admitted, since no explicit information was given in the text. Three Italian regions recorded a high frequency level: Abruzzo (*N* = 24, 35.8%), Apulia (*N* = 20, 29.8%), and Latium (*N* = 10, 14.9%). The remaining cases occurred in: Tuscany (*N* = 5, 7.5%), Campania (*N* = 3, 4.5%), Molise (*N* = 2, 3.0%), Sicily (*N* = 2, 3.0%) and Liguria (*N* = 1, 1.5%). Results are shown in Fig. 1.

### Source of infection

The source of infection was reported in 71 cases (97.2%). In most of the patients (*N* = 48, 65.7%), the infection was related to the consumption of raw or undercooked anchovies. In 11 other cases (15.1%), it was related to the consumption of generically defined “anchovies or sardines” and in one case (1.4%) to sardines (Appendix B). In the remaining 11 cases (15.1%), the source of infection was only mentioned as raw fish or seafood.

### Parasite detection and identification method

The parasite was detected by endoscopy (*N* = 37, 51.4%) or by laparotomy (*N* = 35, 48.6%). Results of the multiple logistic regression showed that only the location (gastric) was significantly associated with the detection method (endoscopy) (OR = 35.70; 95% confidence interval 5.3–293.3), whereas the time of symptom onset was not statistically associated, probably due to the small sample size in each category of this covariate.

In most of the cases, the parasite was generically identified as *Anisakis* spp. (*N* = 42, 57.5%), histologically or by microscopy. In 12 cases (16.4%), the identification achieved by histology or light and scanning microscopy was *A. simplex* (*sensu latu*). In all the 21 cases in which specific molecular methods were used, the identified parasite was *A. pegreffii.* Details are reported in Appendix B.

### Site of infection and number of larvae

The site of infection was classified on the basis of the parasite localisation: gastric (including the oesophagus), or intestinal tract, and ectopic. As regards the gastric localisation, in 32 of the patients the larva was found in the stomach (43.8%) and in 1 in the oesophagus (1.4%), while it was located in the intestine in 31 patients (42.5%). The intestinal location affected the ileum (*N* = 14), the colon (*N* = 11), the caecum (*N* = 4) and the jejunum (*N* = 2). Ectopic locations were found in 9 patients (12.3%), more precisely the peritoneum (*N* = 8) and the spleen (*N* = 1) (Appendix B).

With the exception of two cases (described in [75] and [40]) in which 2 and 3 larvae were endoscopically removed, a single larva was always found.

### Symptoms and time between the ingestion of infected food and the onset of symptoms

In the large majority of the cases, the patients presented with acute or chronic abdominal pain of variable intensity (*N* = 68: 93.1%). Abdominal pain was frequently associated with nausea (16 cases), less frequently with diarrhoea (*N* = 2), fever (*N* = 1) or constipation (*N* = 1). In 10 cases, it was accompanied by an allergic reaction (3 cases reported in [71] and 5 in [70]; 1 case described by Fazii et al. [47] and 1 case described by D’Amelio et al. [35]; see also Serodiagnosis in the analysed cases section). In one case, chest discomfort was the only symptom [96], and in two cases, no symptoms were present [47]. In another case, the patient showed only rectal bleeding, which was first attributed to a possible haemorrhoidal problem, but a larva was found on the colon mucosal surface after colonoscopy [3] (Appendix B).

As regards symptom onset, in most of the cases (69.9%) symptoms were acute (occurring within 24 h, *N* = 28) or sub-acute (occurring within one week, *N* = 23). A highly significant statistical association (*p* < 0.001) was observed between the larval localisation and the time of symptom onset: gastric cases presented symptoms within 24 h of the ingestion of a food at risk, while onset was subacute or chronic in extra-gastric locations. In 14 other cases, the precise timing of consumption of food at risk was unknown, but the patients were habitual consumers of raw or undercooked fish. Most of these cases were extra-gastric (4 omentum, 3 ileum, 2 colon, 1 caecum, 1 spleen), while 3 were gastric. In only one case, the consumption occurred a few months before, and in two cases the time was not known or specified (Appendix B).

### Treatment and outcome

In 36 cases (49.3%), the treatment was the removal of the larva or of the lesion with the larva by biopsy forceps during endoscopy. Overall, 29 of these cases were gastric and a highly significant statistical association (*p* < 0.001) was found between endoscopic removal of the larva and gastric localisation. In all cases, this was the only treatment performed. In the 7 remaining cases treated by endoscopy, the site of infection was the colon (6) or the oesophagus (1).

In 36 cases (49.3%), the patients underwent surgery for the removal of the lesion, generally a mass or an ulcer. In one case, the treatment was not available. In 34 of the cases treated with surgery (94.4%), the localisation of the larva was extra-gastric (25 intestinal localisations, 8 mesentery/omentum, 1 spleen), while only 2 patients who underwent surgical treatment had gastric anisakiasis. The statistical analysis showed a highly significant association (*p* < 0.001) between the surgical treatment and an extra-gastric site of infection of the larva. On the contrary, due to the non-specificity of symptoms, no significant association was observed between symptoms and time of onset nor with symptoms and treatment. In all the cases, the detection method (endoscopy/laparotomy) corresponded to the treatment (endoscopic removal/surgical treatment).

In most of the analysed cases (*N* = 47, 64.4%), no data on the outcome were reported and in one case, follow-up was in progress. When the post-treatment course was described, it was always regular and uneventful. In particular, a regular post-operative course was described for 10 of the patients who underwent surgery, and for 10 patients who were treated by endoscopy.

### Occurrence of allergic reactions

An allergic reaction was described in 10 cases (13.7%): 1 reported in D’Amelio et al. [35], 1 in Fazii et al. [47], 3 in Mattiucci et al. [71] and 5 in Mattiucci et al. [70]. In all cases, the symptoms were strictly associated with the ingestion of contaminated fish (confirmed by the subsequent retrieval of a larva). In 8 cases, high titres of IgE were found. In 5 cases, in particular (Mattiucci et al. [70]) iCAP was >100 kUA/L and IgE-WB positive to excretory/secretory (ES) antigens of *A. pegreffii* (*Ani s 1-like, Ani s 7-like, Ani s 13-like*). The symptoms varied from localised or generalised oedema to urticaria, and were generally associated with abdominal pain. In addition, in one article reporting 11 cases [86], it was mentioned that recurrent oedema in the form of transient swelling, erythema, rashes or widespread itching occurring before or during the development of the infection were rarely observed, again associated with epigastric pain.

### Serodiagnosis in the analysed cases

The serodiagnosis was performed in 26 cases (35.6%), using the UniCAP or ImmunoCAP (Phadia) methods (*N* = 16), the Lopharma method (*N* = 3) and/or immunoblotting (*N* = 13). In 4 cases, the diagnostic method was not mentioned. Only one article reported seronegativity (using an ImmunoCAP System, [3]), while for the remaining cases serological tests were not mentioned. Details on data for the serodiagnosis are reported in Table 2. As regards the interpretation of the results obtained with different methods, in a recent study, IgE response was analysed by an immunoblotting (WB) assay, using both ES antigens and crude extract (CE) of *A. pegreffii* larvae, and the results were compared with those achieved by the conventional immunological method for *Anisakis* allergy (i.e. immunoCAP). Although WB appeared to be more specific, iCAP and WB exhibited a high concordance value (κ = 1.00) when the iCAP value was <0.35 (negative result) and >50.0 (positive result) [70].

**Table 2.** Available data on the serodiagnosis from the included cases.

Reference	Seropositivity and detection method
Fazii et al. [47]	High titre of anti-*Anisakis* antibodies (IgE 13.80 U RAST – Class 4/IgG: 6.3 E)
	High titre of anti-*Anisakis* antibodies
	High titre of anti-*Anisakis* antibodies
Aloia et al. [3]	Serum immunoglobulin (IgM, IgA, IgG and total IgE) levels were within the normal range. Specific IgE to *A. simplex*, using an ImmunoCAP System (Phadia), was negative (<0.35 kU/L)
Pontone et al. [96]	Positive anti-*Anisakis* larvae immunoglobulin G antibody
Mattiucci et al. [71]	Total IgE: 1,479; IgE-As (ImmunoCAP ISAC diagnostic test, Phadia, Uppsala, Sweden): >100; WB positive for *Ani s1* (24 kDa)
	Total IgE: 2,180; IgE-As (ImmunoCAP ISAC diagnostic test, Phadia, Uppsala, Sweden): >100; WB positive for *Ani s1* (24 kDa)
	Total IgE: 4727; IgE-As (ImmunoCAP ISAC diagnostic test, Phadia, Uppsala, Sweden): >100
	Total IgE: 511; IgE-As (ImmunoCAP ISAC diagnostic test, Phadia, Uppsala, Sweden): 21.2
	Total IgE: 2,062; IgE-As (ImmunoCAP ISAC diagnostic test, Phadia, Uppsala, Sweden): 89.3
Mattiucci et al. [70]	ImmunoCAP 50–100 kUA/L, WB positive to ESP antigens of *A. pegreffii*: *Ani s 1-like, Ani s 7-like, Ani s 13-like*
	ImmunoCAP > 100 kUA/L, WB positive to ESP antigens of *A. pegreffii*: *Ani s 1-like, Ani s 7-like, Ani s 13-like*
	ImmunoCAP > 100 kUA/L, WB positive to ESP antigens of *A. pegreffii*: *Ani s 1-like, Ani s 7-like, Ani s 13-like*
	ImmunoCAP > 100 kUA/L, WB positive to ESP antigens of *A. pegreffii*: *Ani s 1-like, Ani s 7-like, Ani s 13-like*
	ImmunoCAP > 100 kUA/L, WB positive to ESP antigens of *A. pegreffii*: *Ani s 7-like, Ani s 13-like*
Mattiucci et al. [75]	WB assay IgE, IgG4 reactivity *versus Ani s 13-like* and *Ani s 7-like*
	WB assay only IgG reactivity *versus Ani s 13-like* and *Ani s 7-like*
	WB assay, IgE and IgG reactivity *versus Ani s 13-like*, *Ani s 7-like* and *Ani s 1-like*

## Discussion

### Literature search and selection of cases

Despite the strict selection applied, the number of cases detected is higher than the number indicated in other recent Italian studies [34, 38]. In addition, although great efforts were made during the bibliographic investigation, it is known that other cases (not described in the literature) are also reported annually by the health authorities, mainly in regions of Central and Southern Italy [38]. However, access to these data is generally not public and a database at the national level is lacking.

The low number of documented cases is in contrast with the frequent occurrence of these parasites in a wide number of Mediterranean fish species [60, 68], including those consumed raw or marinated, suggesting that human infections with *Anisakis* spp. in Italy might still be significantly underestimated [68, 76], due to the high frequency of self-limiting infections [38], to underreporting, and to missed or incomplete diagnosis. In this regard, it should also be stressed that the selection applied in this study led to the exclusion of 34 “suspected” cases (27 from the excluded cases and 7 from the included cases). Other authors already pointed out that many more cases may be suspected if serologically positive subjects, but without detection of parasites, are considered [90]. However, in order to estimate correct epidemiological data, a precise etiological identification should always be sought (see Parasite detection and identification method section). An additional factor that can contribute to the underestimation of cases due to missed or incomplete diagnosis is the general low level of knowledge of seafood parasitology among Italian medical doctors. In fact, in Italy as in other European countries, teaching parasitology to medical students is met with a decreasing level of interest [19]. The same factors causing underestimation of *Anisakis* spp. are likely to also influence underestimation of imported infections by *Pseudoterranova* spp. in Italy.

### Geographical distribution

The infection appears to be more frequent in coastal areas, where there is a higher tendency to consume seafood. The results of the present retrospective study are in agreement with other studies claiming that the disease is more widespread in the regions of Southern and Central Italy (Abruzzo, Molise, Campania, Puglia, and Sicily) [34]. On the contrary, no marked geographical differences were found in the recent study conducted in France [119]. However, the presence of specialised researchers or centres in specific regions may represent a bias in the geographical distribution of cases, influencing the attention to specific diseases which may be more frequently described in such areas. This phenomenon has already been observed by other authors [110] and it had already been hypothesized for the Abruzzo region, where the existence of a specialised group of experts likely favoured the diagnosis of a higher number of cases [40]. In this respect, the continuous education of physicians and the creation of dedicated centres distributed throughout the country appears important for correct diagnosis and for the acquisition of up-to-date epidemiological data. An example is the Apulia region where, given the high frequency of the disease, also confirmed by the results of the present study, a Technical Working Group for the “prevention of *Anisakis* spp. in fishery products” was created in 2011, with the aim of implementing a system of surveillance in coordination with the Regional Epidemiological Observatory (https://www.sanita.puglia.it/archivio-news_det/-/journal_content/56/20182/prevenzione-dell-anisakiasi-da-consumo-di-prodotti-della-pesca).

### Source of infection

It is widely recognised that the source of human anisakiasis relates to the geographical areas and to different eating habits [110]. Several commercial fish species distributed in European markets are at risk of carrying zoonotic anisakid nematodes [43, 68]. Raw or pickled herring, hake, mackerel, anchovy and cod are known to be important infection sources in other European countries, such as the Netherlands and Spain [10, 102, 118]. Spain, in particular, is considered to have the highest incidence of anisakiasis in Europe, predominantly through consumption of the traditional marinated dish “anchovies in vinegar”. However, the actual human anisakiasis burden is unknown due to the scarcity of epidemiological data [13, 43]. Anchovies (*Engraulis encrasicolus)* are traditionally consumed raw following simple home-made preparations (in lemon or vinegar, according to the regional/local traditional recipes, i.e., “marinated anchovies”) also in Italy and in Croatia [33, 79]. Salmon was shown to be the food source most frequently involved in human cases in France, followed by anchovies [119].

On the basis of the data analysis, anchovies are confirmed as the main species at risk in Italy, in agreement with [71, 74–76]. In Italy, *E. encrasicolus* is the main fished species by weight, corresponding to 25–35% of the total catches of marine fishes between 2010 and 2014 (http://www.fao.org/fishery/topic/16140/en). As mentioned, this fish species is frequently consumed in recipes traditionally prepared without thermal processing, such as marinated anchovies. Accordingly, in the vast majority of the cases analysed here, it was reported that the fish had been consumed as marinated or pickled (*N* = 53, 72.6%).

Besides anchovies, pilchards (or sardines) were also indicated as a possible source of infection in the literature analysed. In 11 cases, in fact, the infection was related to the consumption of generically indicated “anchovies or sardines”, and in one case to sardines. Epidemiological data regarding the two fish species are available in the literature. The occurrence of *Anisakis* spp. in anchovies from the Mediterranean Sea has been reported in a number of studies [6, 27, 31, 32, 38, 53, 78, 80, 94, 100, 106] with variable infection levels. A summary of most of the data deriving from these surveys is reported in Cipriani et al. [33]. This latter study conducted an extensive survey on anchovies in different Mediterranean fishing grounds, showing that levels of infection with *A. pegreffii* significantly varied in relation to areas and, to a lesser extent, fish size. In particular, the highest levels of infection were found in the Central (70.8%) and South Adriatic Sea (55.8%), while anchovies from Southern Sicily, the Ionian and Alboran Seas were uninfected [33]. High *Anisakis* spp. prevalence values (reaching in some batches 83% and 29% in the viscera and flesh, respectively) were also found in the north-east Atlantic [102]. Similarly, differences in the infection level of *Anisakis* spp. in European pilchards (sardines) from the Mediterranean Sea have been reported [27, 78, 81, 94, 106], ranging from 0% in the Ligurian Sea [106], to 28.3% along the Atlantic Spanish coast [81]. In a recent study, the prevalence (varying from 0 to 44.9%, average 12.2%) was confirmed to be highly influenced by both geographic location and host size. In this case, the highest prevalence (44.9%) was recorded in western parts of the Mediterranean, in particular off west Sardinia [22]. Sardines from the Atlantic showed comparatively high *Anisakis* spp. prevalence in both viscera (approximately 62%) and flesh (17%) in another recent survey [60].

Importantly, in both anchovies and sardines, *Anisakis* spp. larvae were also found in the muscle, although with lower prevalence values compared to viscera [22, 33, 60].

Thus, given the variable prevalence values found in anchovies and in sardines, it is difficult to hypothesize how many ambiguous cases could be attributed to one species rather than to the other. In relation to this, we should also note that many consumers are not able to clearly distinguish anchovies and sardines, leading to a certain degree of confusion between the two species, especially if marinated.

As regards dish preparation, few details were given and only the studies of Fumarola et al. [49] and Mattiucci et al. [70] specified that marinated/pickled anchovies had been domestically prepared, while in another study it was specified that the dish had been prepared from raw fresh anchovies [71]. Nematodes are highly resistant to traditional marinating methods, surviving for periods of a few days up to several weeks, depending on the concentration of salt, acetic acid, and marinating times [1, 4, 56]. In the traditional marinating process, the fish is left in a solution of vinegar and salt for <24 h. However, Sánchez-Monsalvez et al. [105] reported that the death of all larvae in fillets exposed to vinegar did not occur until day 13. Therefore, preventive freezing of such products is compulsory by law in order to reduce the parasite hazard [36]. The industrial production of salted or marinated anchovies generally assures devitalisation of the larvae [4, 97], and only dead larvae were found in two recent studies on industrially [52] or professionally [112] marinated anchovies.

Although Italian legislation directed at domestic preparation states that:

*In case of consumption as raw, marinated or not fully cooked fish, the product must first be frozen for at least 96 hours at −18 °C in a domestic freezer marked with three or more stars [* *59* *]*

fish prepared at home still represents a particular challenge for risk prevention, as also observed by other authors [60]. In fact, domestic preparation is often conducted without such previous freezing [60], since it can affect the taste characteristics of the products [4], or by freezing for an inadequate period of time [90], allowing survival of the larvae and involving a transmission risk. Interestingly, a significantly positive correlation between the *post-mortem* migration of *A. pegreffii* in anchovy and incorrect storage temperature and time was demonstrated. In particular, the increase of infection values with *A. pegreffii* in anchovy fillets was statistically positively related to the increase of the temperature and time of storage [32]. Larval migration during storage at 4 °C was also observed by Šimat et al. [109], as well as an influence of biogenic amines and pH. Moreover, it was recently shown that *Anisakis* spp. may present some freezing tolerance, allowing partial survival when submitted to domestic freezing, owing to insufficiently low temperatures, temperature inhomogeneity, and different freezing rates [104].

Among the 11 cases for which no reference to a fish species was made, 2 of them specified that the source food was sushi [40, 84]. Although sushi and sashimi are a food at risk for transmitting anisakidosis, various marine fish species that are preferentially served in Japanese restaurants and sushi bars in Japan such as tuna, yellow tail, red snapper, salmon, and flatfish/flounder are less contaminated or even free of *Anisakis* larvae. In contrast, other popular and cheaper marine fish, such as cod, herring, mackerel, and squid that are mainly consumed at home or at local restaurants in Japan, tend to be heavily infected with *Anisakis* spp. larvae [86].

Similarly, the species most frequently used in sushi preparation in Italy like salmon, gilt-head seabream, sea bass and tuna [8, 9], are rarely infected by anisakids. In fact, with the exception of tuna, they are farmed species for which the parasitological risk is usually lower [7, 43, 68, 92]. This might explain why, despite the fact that culinary habits have changed in Italy and that, similarly to other European countries, there has been a strong increase in sushi/sashimi consumption, no increase of published cases during the studied period was observed. Despite this, *Anisakis* spp. might still represent a risk if infected fish products are served in sushi restaurants, given the low awareness related to the correct use of preventive freezing techniques of many operators demonstrated in a survey in Florence [8]. This might be the origin of the case reported by Palma et al. [89] where the source of infection was identified as raw anchovies consumed at a sushi restaurant five days before the endoscopy.

### Parasite detection and identification method

To date, nine species belonging to the genus *Anisakis* have been identified worldwide [76]. However, only two of them, *A. simplex* (*s.s.*) and *A. pegreffii,* have been reported as responsible for infections in humans [71, 76]. Unambiguous identification of anisakids is an essential requirement for conducting epidemiological surveys [61]. In this light, more accurate diagnosis of human anisakidosis by applying sensitive, rapid and specific molecular methodologies, ideally combined with serodiagnostics such as immunoblotting, would greatly improve knowledge on anisakid epidemiology [68]. In this study, in all the 16 cases for which a molecular diagnosis was available, *A. pegreffii* was found to be the causative agent of anisakiasis. These data confirm that in the Mediterranean region, the zoonotic risk is mainly associated with the presence of this species [73, 76, 79]. In fact, *A. pegreffii* frequently occurs in various fish and squid species from the Mediterranean Sea [72] and it is the species most frequently involved in zoonotic infections in Italy [35, 68, 71, 74–76]. It should be noted that the first Italian case of invasive infection with *Pseudoterranova decipiens* was recently described in a woman from Sicily. Unfortunately, it was not possible to clearly identify the source of infection likely due to imported infected fish. The parasite identification was confirmed by sequence analysis of mtDNA cox2 gene [28], further highlighting the importance of pursuing molecular diagnosis.

In the cases of histological examination, the diagnosis was based on the morphological features of the nematode larva (such as the presence of polymyarian muscle cells in a transverse section, shape of the lateral chords, oesophagus with a triangular lumen). The presence of the detected larva was generally associated with an eosinophilic infiltrate or granuloma [5, 15, 26, 64, 67, 82–84, 90, 91]. Pathologists should consider the possibility of anisakid infection when facing an eosinophilic granuloma of the digestive tract, the mesentery, or the peritoneum. Several sections of the granuloma must be performed in seeking sections of the nematode, since in some cases the presence of the larva is not particularly obvious and may be overlooked [82, 90]. However, currently, it is also possible to perform molecular diagnosis on a single histological section, based on real-time PCR DNA primer-probe systems [75].

### Site of infection and number of larvae

A confirmed aetiological diagnosis is based on directly finding the nematode larva in the relative organ; thus gastric anisakidosis is generally more easily detected, while the diagnosis in the case of intestinal or ectopic locations is often more challenging [62]. The present results show that in the cases included in the study, gastric and intestinal localisations had very similar frequencies, only slightly higher for gastric lesions. Similarly, in a retrospective case series study conducted on 83 cases in Tokyo, Japan, 47% of patients presented gastric anisakiasis and 53% small intestinal anisakiasis [114]. However, other authors state that in Japan, the acute gastric form prevails (95%), while in Europe the chronic intestinal form seems to be more frequent [82, 108].

Beside unusual gastrointestinal localisation such as the colon, caecum and oesophagus [5, 12, 39], rarely, anisakid larvae have been found even in other extra-intestinal organs such as larynx, lungs, lymph nodes, uterus, ovaries, spleen, liver and pancreas [58, 90, 98].

In most of the cases, one larva was found, in agreement with observations of other authors [87]. It is known that one single larva can be responsible for clinical symptoms [63].

### Symptoms and time between the ingestion of infected food and the onset of symptoms

Epigastric pain, frequently associated with nausea and vomiting, was the most frequently observed symptom in the present retrospective analysis, in agreement with recent studies [110, 114]. These observations support the suggestion of some authors who consider anisakiasis as a misdiagnosed and underestimated cause of acute abdomen disorders [25]. In particular, anisakiasis should be considered when the cause of abdominal pain cannot be determined by initial assessment [114].

In general, symptoms were not specific and the co-existence of other gastroenteric disorders complicated their interpretation. In these cases, indirect serological tests (skin-prick test and specific IgE) but also non-specific parameters, when present (leucocytosis, hypersedimetry, eosinophilia), can provide useful diagnostic indications [82].

As regards symptom onset, the highly significant statistical association between the larval localisation and the time of symptom onset observed for the cases included here, with gastric cases presenting symptoms within 24 h from the ingestion of a food at risk, and subacute or chronic onset in extra-gastric locations, was observed by Takabayashi et al. [114]. Similarly, in most of the 645 Korean cases reviewed by Sohn et al. [110], of which 82.4% presented a gastric localisation, symptoms occurred within 12 h after raw or undercooked fish consumption.

Overall, most of the patients could recall eating raw or undercooked fish, and some of them were habitual consumers. Only in one case, the patient reported not having consumed raw fish, a fact that might be explained considering that not all consumers are aware that marinated fish should actually be considered as raw. This should be kept in mind when investigating the history [47, 114].

### Treatment and outcome

In acute gastric forms, endoscopy represents not only a valid diagnostic tool but also the elective treatment for gastric anisakiasis [23, 114], as also observed in this retrospective study. Many reports included in the study state that the patient had a prompt recovery after removal of the worm by endoscopy [49, 65, 117]. Also Shimamura et al. [108] observed that extraction of the larvae will usually result in prompt symptom resolution. For effective treatment, however, it is important to ensure that there is no remaining larva within the gastric wall. Thorough examination of the stomach is crucial as there is a possibility of multiple infections [87] and it is challenging to identify the larvae especially in the greater curvature [108], the most frequent parasitic location in the organ [87, 107]. For the other forms of anisakiasis, the choice of treatment depends on the specific complications [97]. Since the symptoms of anisakiasis are not pathognomonic, gastric anisakidosis is often misdiagnosed as peptic ulcer, stomach tumour or stomach polyps, while intestinal anisakidosis is often misdiagnosed as appendicitis, ileus, or peritonitis [62, 103, 114]. Pampiglione et al. [90] pointed out that the clinical diagnosis was incorrect in all the 11 cases reviewed in their study. Interestingly, in most of the cases undergoing surgery reviewed here, very often other diseases were suspected (in most of the cases appendicitis) and the diagnosis of anisakiasis was only performed after the histological examination of the sample (Appendix B).

To date, there is no definitive medicinal therapy [108]. Albendazole and ivermectin have been shown to be effective [97]. As concerns the antiparasitic treatment in the cases reviewed here, albendazole was given in one patient with colon localisation treated by endoscopy, while in 3 patients it was associated with surgery. The various compounds and dosages are reported in Appendix B. The use of antiparasitic drugs alone is controversial, since once *Anisakis* spp. larvae have penetrated the thickness of the intestinal wall, though subsequently killed by the drug, they can still cause an antigenic reaction, and may contribute to the development of eosinophilic granuloma [90].

### Occurrence of allergic reactions

Gastrointestinal anisakiasis may be accompanied by IgE-mediated allergic reactions, ranging from urticaria or angio-oedema to anaphylaxis [70, 71, 75]. The condition of patients who develop IgE-mediated allergic reactions and gastrointestinal symptoms simultaneously is generally indicated as “gastro-allergic anisakiasis (GAA)”, an acute allergic reaction with symptoms of hypersensitivity appearing several hours after the ingestion of infected, raw and/or undercooked fish, associated with penetration of larvae into the gastric mucosa [37]. In some cases, an IgE antibody response in individuals with no apparent symptoms has been detected one month after the acute GAA episode [70]. Other reports of allergic reaction related to *Anisakis* infections occurred without larval detection and identification of the parasite, and some patients may only present classic manifestations of IgE-mediated allergy such as urticaria, angio-oedema and anaphylaxis [50]. In fact, *Anisakis* spp. have also been deemed responsible for occupational allergies following skin contact or inhalation of allergens [14] and a high level of *Anisakis*-IgE hypersensitivity in Italian fishery products handlers, fishermen and consumers of fish was reported using the iCAP methodology [77]. However, in these patients, the presence of a larva was not detected. The sensitisation pathway is not completely understood. *Anisakis* sensitisation is most frequently reported from Mediterranean countries, Japan or South Korea and it has been associated with high consumption levels of raw or undercooked fish, since it is believed that a prior infection with live larvae is needed for sensitisation to the parasite allergens. Thus, this differential IgE sensitisation could be related to the higher consumption of raw, undercooked or marinated fish in Mediterranean countries, as opposed to the lower levels of these dishes consumed in North European countries [70].

As a consequence, the cases included in the present study, that always harboured an infective larva, could be at risk of developing sensitisation to *Anisakis* spp. antigens. In fact, it can be supposed that for both *A. pegreffii* and *A. simplex (s.l.)* not only live, but also non-viable larvae or related antigens could be involved in chronic urticarial reactions, despite the mechanism still needing to be discovered [70].

## Conclusions

This is the first retrospective study reviewing epidemiological data and clinical manifestations of the cases of anisakiasis occurring in Italy since its first description. The number of cases of anisakiasis is certainly higher than reported, since many might not be diagnosed or published, and others might heal spontaneously. Our study suggests that human anisakiasis must be taken into account in the differential diagnosis of acute abdominal syndromes, particularly in patients who usually consume raw or undercooked fish. Furthermore, it highlights common aspects between cases and points out limits in diagnosis and treatment. These aspects may be of interest to medical doctors for managing anisakiasis in other countries.

Pathologists should take into account the possibility of this parasitic infection when examining an eosinophilic granuloma of the digestive tract, mesentery or peritoneum. Underestimating this possibility can lead to a considerable delay in diagnosis, and thus in treatment. In addition, clinicians should be encouraged to perform serodiagnosis by using appropriate diagnostic methods and to carry out and report molecular identification of the parasite, to increase knowledge about the association between different *Anisakis* spp. and their pathogenic effects on humans. Specific attention should also be given to the patient history in order to investigate the likely source of infection. These data are often lacking or uncertain but they are essential for better monitoring and control of food-borne parasites using risk assessment tools.

Prophylaxis is based on health education principles aiming at informing consumers on how to manage the infection risks. Adequate health education of consumers with regard to fish preparation at home is thus of the utmost importance. The results of the present review highlight that particular attention should be given to raw marinated anchovies, which are proven to be the main source of human anisakiasis in Italy. Considering the globalisation of the food supply and changes in culinary habits, as well as the possibility that traditional dishes are also consumed by tourists visiting the country, the identification of this source as a food at risk is not limited to a national level. Finally, the importance of this study is also related to the lack of similar data available in the literature.

## Conflict of interest

The authors declare that they have no competing interests.

## Appendices

**Appendix A:** Bibliographic research to identify cases of anisakiasis

	PubMed retrieved	PubMed selected for screening	Google Scholar retrieved	Google Scholar selected for screening
Anisakidae and (human or human cases) and Italy	16	2	536	17
*Anisakis* and (human or human cases) and Italy	60	23	1420	32
Human anisakidosis and Italy	7	4	320	26
Human anisakiasis and Italy	41	22	1630	33
(Human anisakidosis or human anisakiasis) and Italy	43	24	315	25
Human anisakidosis or human anisakiasis and Italy	111	27	315	25

**Appendix B:** Details of the data collected from the included cases.

Reference	Sex	Age	Geographical origin	Source of infection and time between consumption and symptoms insurgence	Parasite species	Method used for larval detection	Identification method	Parasite location	Symptoms	Treatment/outcome
Stallone et al. [113]	F	40	Apulia, Bari	Raw fish products consumed a few hours before	*Anisakis* sp.	n.a.	microscopy	Gastric	Epigastric pain	n.a./n.a
Cancrini et al. [24]	F	38	Sicily, Catania	Habitual consumption of raw fish products	*Anisakis* sp.	Laparatomy	Histology	Omentum	Severe pain in the ileocecal region	Surgery/n.a.
Cancrini et al. [23]	M	50	Sicily, Catania	Raw fish (“*pesce azzurro”*) consumed 10 h before	*Anisakis* sp.	Endoscopy	Microscopy	Gastric	Acute epigastric pain	Removal by endoscopy/n.a.
D’Amelio et al. [35]	F	51	Apulia, Bari	Habitual consumption of raw fish and squids	*A. pegreffii*	Endoscopy	PCR-RFLPs	Gastric	Acute severe epigastralgia, followed 20 days later by generalized urticaria, neck and head paresthesia	Removal by endoscopy/n.a.
Maggi et al. [64]	F	43	Apulia	Unclear, raw fish normally consumed	*A. simplex*	Laparatomy	Histology	Gastric	Epigastric pain	Surgery/no complications
	F	34	Apulia	Non-consumer of raw fish	*A. simplex*	Laparatomy	Histology	Ileum	Abdominal pain	Surgery/no complications
	F	43	Apulia	Pickled anchovies normally consumed	*A. simplex*	Laparatomy	Histology	Jejunal mesentery	Abdominal cramps	Surgery + mebendazole (200 mg/day in 2 doses for 3 days)/follow up in progress
Pampiglione et al. [90]	F	30	Apulia, Bari	Raw marinated sardines or anchovies occasionally consumed	*Anisakis* sp.	Laparatomy	Histology	Ileum	Acute abdominal syndrome	Surgery/n.a.
	F	22	Molise, Campobasso	Raw marinated sardines or anchovies consumed a few days before	*Anisakis* sp.	Laparatomy	Histology	Mesentery	Acute appendicitis	Surgery/n.a.
	F	54	Apulia, Bari	raw marinated sardines or anchovies consumed a few days before	*Anisakis* sp.	Laparatomy	Histology	Omentum	Intestinal occlusion	Surgery/n.a.
	M	37	Apulia, Bari	Raw marinated sardines or anchovies occasionally consumed	*Anisakis* sp.	Laparatomy	Histology	Omentum	Gastric ulcer	Surgery/n.a.
	F	44	Molise, Campobasso	Raw marinated sardines or anchovies consumed 4 days before	*Anisakis* sp.	Laparatomy	Histology	Omentum	Intestinal occlusion	Surgery/n.a.
	F	60	Apulia, Bari	Raw marinated sardines or anchovies habitually consumed	*Anisakis* sp.	Laparatomy	Histology	Epiploic appendix	Colon neoplasia	Surgery/n.a.
	M	48	Apulia, Brindisi	Raw marinated sardines or anchovies consumed 8h before	*Anisakis* sp.	Laparatomy	Histology	Ileum	Intestinal occlusion	Surgery/n.a.
	F	41	Apulia, Brindisi	Raw marinated sardines or anchovies consumed 10 days before	*Anisakis* sp.	Laparatomy	Histology	Ileum	Acute appendicitis	Surgery/n.a.
	M	56	Apulia, Bari	Raw marinated sardines or anchovies consumed few hours before	*Anisakis* sp.	Laparatomy	Histology	Gastric	Acute abdominal syndrome	Surgery/n.a.
	F	86	Apulia, Bari	Raw marinated sardines or anchovies habitually consumed	*Anisakis* sp.	Laparatomy	Histology	Spleen	Acute abdominal syndrome	Surgery/n.a.
	F	56	Apulia, Brindisi	Raw marinated sardines or anchovies consumed 6 h before	*Anisakis* sp.	Endoscopy	Microscopy	Gastric	Epigastric pain	Removal by endoscopy/n.a.
Caramello et al. [25]	F	31	Liguria, Savona (H)	Raw or undercooked fish consumed seven days before admission	*Anisakis* sp.	Laparatomy	Histology	Ileum	Severe abdominal colicky pain, accompanied by diarrhoea and vomiting	Surgery/no complications
Moschella et al. [83]	F	37	Latium, Rome (H)	Raw marinated anchovies consumed 20 days before	*Anisakis* type 1	Laparatomy	Histology	Colon	Acute abdominal pain, nausea and emesis	Surgery + 200 mg mebendazole b.i.d. for 3 days/no complications
De Nicola et al. [39]	M	40	Abruzzo, Chieti (H)	Raw anchovies consumed few days before	*A. simplex*	Laparatomy	Histology	Caecum	Abdominal pain, nausea and fever	Surgery/no complications
Montalto et al. [82]	M	41	Latium, Rome (H)	Marinated sardines about 7 days before and regularly	*A. simplex*	Laparatomy	Histology	Ileum	Partial intestinal obstruction evolving into acute abdomen	Surgery with resection of the right colon and the terminal ileum. 2 wrong diagnosis: Chron and eosinophilic enteritis. Treatment with pyrantel pamoatem, paromomycin, then metronidazole and low doses of steroids when finally anisakiasis was diagnosed (2 years later)/no complications
Pellegrini et al. [91]	M	33	Tuscany, Siena	Pickled anchovies consumed a few days before	*A. simplex*	Laparatomy	Histology	Jejunum	Diffuse abdominal pain accompanied by vomiting	Surgery/no complications
Fazii et al. [47]	F	32	Abruzzo, Chieti	Marinated anchovies consumed in the preceeding days	*Anisakis* sp.	Endoscopy	Microscopy	Gastric	Epigastric pain	Removal by endoscopy/n.a.
	M	66	Abruzzo, Pescara	Marinated anchovies consumed in the preceeding days	*Anisakis* sp.	Endoscopy	Microscopy	Gastric	Gastroscopy for control	Removal by endoscopy/n.a.
	M	45	Abruzzo, Pescara, Popoli	Marinated anchovies consumed in the preceeding days	*Anisakis* sp.	Endoscopy	Microscopy	Colon	Colonscopy for control	Removal by endoscopy/no complications (diet without seafood and gloves during work)
Avellino et al. [12]	F	49	Latium, Rome (H)	Not reported	*A. pegreffii*	Endoscopy	PCR-RFLP	Esophagus	Not described	Removal by endoscopy/n.a.
Ugenti et al. [117]	F	51	Apulia, Bari (H)	Raw anchovies consumed 2 h before	*A. simplex* complex	Endoscopy	Light and scanning electric microscopy	Gastric	Very severe epigastric pain, vomiting	Removal by endoscopy/prompt recovery, no complications
	F	46	Apulia, Bari (H)	Raw anchovies consumed 12 h before	*A. simplex* complex	Endoscopy	light and scanning electric microscopy	Gastric	Severe epigastric pain without nausea or vomiting	Removal by endoscopy/prompt recovery, no complications
	F	61	Apulia, Bari (H)	Raw anchovies consumed 12 h before	*A. simplex* complex	Endoscopy	light and scanning electric microscopy	Gastric	Violent epigastric pain	Removal by endoscopy/prompt recovery, no complications
Fumarola et al. [49]	F	49	Apulia, Bari	Homemade raw pickled (vinegar) anchovies consumed 6 h before	L3 larva of the genus *Anisakis* on the basis of morphological criteria. *A. pegreffii* by PCR-RFLP	Endoscopy	Microscopy + PCR-RFLP	Gastric	Bloody vomiting and severe gastric pain	Removal by endoscopy/prompt recovery, no complications
	F	59	Apulia, Bari	Regular eater of undercooked fish	L3 larva of the genus *Anisakis* on the basis of morphological criteria. *A. pegreffii* by PCR-RFLP	Endoscopy	Microscopy + PCR-RFLP	Gastric	Gastric pain and meteorism	Removal by endoscopy/n.a.
Marzocca et al. [67]	M	33	Tuscany, Siena	Marinated anchovies consumed in the preceeding days	*A. simplex*	Laparatomy	Histology	Jejunum	Severe upper abdominal pain, nausea and vomiting, without fever	Surgery/no complications
Aloia et al. [3]	M	32	Campania, Salerno (H)	Raw marinated anchovies consumed three days before the colonoscopy	*A. simplex*	Endoscopy	Microscopy	Colon	Rectal bleeding with no other symptomatology	Removal by endoscopy + albendazole (400 mg b.i.d. for 4 days)/no complications (diet without seafood)
Mattiucci et al. [74]	M		Latium, Viterbo	Raw anchovies consumed 2 months before	*A. pegreffii*	Laparatomy	DNA extraction technique from paraffin-embedded tissue, followed by PCR (*cox 2)*	Caecum	Acute abdominal pain in the lower right quadrant, nausea and emesis	Surgery/n.a.
De Rosa [40]	F	19	Abruzzo, Chieti, Guardiagrele	Habitual consumption of marinated anchovies	*Anisakis* sp.	Laparatomy	Histology	Caecum	Acute abdomen	Surgery/n.a.
	M	38	Abruzzo, Pescara	Habitual consumption of marinated anchovies	*Anisakis* sp.	Laparatomy	Histology	Ileum	Acute abdomen	Surgery/n.a.
	M	32	Abruzzo, Pescara	Marinated anchovies consumed 7 days before	*Anisakis* sp.	Laparatomy	Histology	Ileum	Acute abdomen	Surgery/n.a.
	M	39	Abruzzo, Chieti, Ortona a Mare	Habitual consumption of marinated anchovies	*Anisakis* sp.	Laparatomy	Histology	Ileum	Acute abdomen	Surgery/n.a.
	M	51	Abruzzo, Pescara	Habitual consumption of marinated anchovies	*Anisakis* sp.	Laparatomy	Histology	Colon	Acute abdomen	Surgery/n.a.
	F	35	Abruzzo, Chieti, Ortona a Mare	Marinated anchovies consumed a few days before	*Anisakis* sp.	Laparatomy	Histology	Ileum	Acute abdomen	Surgery/n.a.
	M	40	Abruzzo, Chieti	Marinated anchovies consumed a few days before	*Anisakis* sp.	Laparatomy	Histology	Caecum	Acute abdomen	Surgery/n.a.
	M	67	Abruzzo, Chieti	Habitual consumption of marinated anchovies	*Anisakis* sp.	Laparatomy	Histology	Colon	Light abdominal pain	Surgery/n.a.
	F	27	Abruzzo, Chieti, Vasto	Marinated anchovies consumed a few days before	*Anisakis* sp.	Laparatomy	Histology	Ileum	Abdominal pain	Surgery/n.a.
	M	59	Abruzzo, Chieti, Lanciano	Marinated anchovies consumed 24 h before	*Anisakis* sp.	Endoscopy	Microscopy	Colon	Acute abdomen	Removal by endoscopy/n.a.
	F	38	Molise, Montecilfone (CB)	Marinated anchovies (only one anchovy) consumed 12 h before	*Anisakis* sp.	Laparatomy	Histology	Ileum	Acute abdomen	Surgery/n.a.
	F	45	Abruzzo, Teramo	Marinated anchovies a few days before	*Anisakis* sp.	Endoscopy	Microscopy	Colon	Abdominal pain	Removal by endoscopy/n.a.
	F	44	Abruzzo, Teramo, Campli	Marinated anchovies consumed 4 h before	*Anisakis* sp.	Endoscopy	Microscopy	Gastric	Epigastric pain	Removal by endoscopy/n.a.
	M	30	Abruzzo, Chieti, Francavilla al Mare	Marinated anchovies consumed 12 h before	*Anisakis* sp.	Endoscopy	Microscopy	Gastric	Epigastric pain	Removal by endoscopy/n.a.
	F	28	Abruzzo, Chieti	Sushi consumed a few days before at a restaurant	*Anisakis* sp.	Laparatomy	Histology	Mesentery	Acute abdomen	Surgery/n.a.
Pontone et al. [96]	F	55	Latium, Rome (H)	Marinated anchovies consumed 12 h before	*Anisakis* sp.	Endoscopy	Microscopy	Gastric	Acute chest discomfort, no abdominal pain	Removal by endoscopy/prompt recovery, no complications
Mattiucci et al. [71]	n.a.	n.a.	Campania, Benevento	Raw fresh marinated anchovies consumed 2 h before	*A. pegreffii*	Endoscopy	PCR (cox II, ITS region)	Gastric	Epigastric pain, edema of oral mucosa	Removal by endoscopy/n.a.
	n.a.	n.a.	Abruzzo, Pescara	Raw fresh marinated anchovies consumed 24 h before	*A. pegreffii*	Endoscopy	PCR (cox II, ITS region)	Gastric	Epigastric pain, urticaria, generalized edema	Removal by endoscopy/n.a.
	n.a.	n.a.	Abruzzo, Pescara	Raw fresh marinated anchovies consumed 24 h before	*A. pegreffii*	Endoscopy	PCR (cox II, ITS region)	Gastric	Epigastric pain	Removal by endoscopy/n.a.
	n.a.	n.a.	Abruzzo, Pescara	Raw fresh marinated anchovies consumed 1 day before	*A. pegreffii*	Endoscopy	PCR (cox II, ITS region)	Gastric	Epigastric pain, digestive symptoms	Removal by endoscopy/n.a.
	n.a.	n.a.	Abruzzo, Pescara	Raw fresh marinated anchovies consumed 1 day before	*A. pegreffii*	Endoscopy	PCR (cox II, ITS region)	Gastric	Epigastric pain	Removal by endoscopy/n.a.
	n.a.	n.a.	Latium, Rome	Raw fresh marinated anchovies consumed 1 day before	*A. pegreffii*	Endoscopy	PCR (cox II, ITS region)	Gastric	Epigastric pain, urticaria	Removal by endoscopy/n.a.
	n.a.	n.a.	Tuscany, Pisa	Raw fresh marinated anchovies consumed 2 days before	*A. pegreffii*	Endoscopy	PCR (cox II, ITS region)	Gastric	Epigastric pain, vomiting	Removal by endoscopy/n.a.
	n.a.	n.a.	Campania, Naples	Raw fresh marinated anchovies consumed 2–3 h before	*A. pegreffii*	Endoscopy	PCR (cox II, ITS region)	Gastric	Epigastric pain	Removal by endoscopy/n.a.
Mumoli and Merlo [84]	M	56	Tuscany, Livorno (H)	Sushi and sashimi consumed at a restaurant a month before	*A. simplex*	Endoscopy	Histology	Colon	Nausea and colicky pain in the lower left abdominal quadrant	Removal by endoscopy/n.a.
Andrisani et al. [5]	F	56	Latium, Rome (H)	Raw fish (time of consumption unknown)	*Anisakis* sp.	Endoscopy	Histology	Colon	Abdominal pain, nausea, vomiting and diarrhoea	Surgery/n.a.
Baron et al. [15]	M	20	Not clear (Campania or Sicily)	Raw anchovies consumed 7 days before the symptoms	*Anisakis* sp.	Laparatomy	Histology	Ileum	Abdominal pain and vomiting	Surgery/no complications
Mariano et al. [65]	F	53	Latium, Rome	Marinated anchovies consumed the night before onset of gastric symptoms	*Anisakis* sp.	Endoscopy	Histology	Gastric	Intense stomach ache associated with aspecific chest pain	Removal by endoscopy/prompt recovery, no complications
Carbotta et al. [26]	M	61	Apulia, Bari	Raw fish consumed 3 days before admission	*Anisakis* sp.	Laparatomy	Histology	Ileum	Abdominal pain, vomit and constipation	Surgery/no complications
Mattiucci et al [70]	n.a.	n.a.	n.a.	Marinated anchovies	*A. pegreffii*	Endoscopy	PCR (COII)	Gastric	Gastro-allergic anisakiasis	endoscopy/n.a.
	n.a.	n.a.	n.a.	Marinated anchovies	*A. pegreffii*	Endoscopy	PCR (COII)	Gastric	Gastro-allergic anisakiasis	endoscopy/n.a.
	n.a.	n.a.	n.a.	Marinated anchovies	*A. pegreffii*	Endoscopy	PCR (COII)	Gastric	Gastro-allergic anisakiasis	endoscopy/n.a.
	n.a.	n.a.	n.a.	Marinated anchovies	*A. pegreffii*	Endoscopy	PCR (COII)	Gastric	Gastro-allergic anisakiasis	endoscopy/n.a.
	n.a.	n.a.	n.a.	Marinated anchovies	*A. pegreffii*	Endoscopy	PCR (COII)	Gastric	Gastro-allergic anisakiasis	endoscopy/n.a.
Mattiucci et al. [75]	F	n.a.	Abruzzo, Pescara	Home-made raw marinated anchovies	*A. pegreffii*	Endoscopy	PCR (COII and EF1 α-1 nDNA region)	Gastric	Epigastric pain and nausea	Removal by endoscopy/prompt recovery, no complications
	M	58	Latium, Rome	Home-made raw marinated anchovies consumed a week before, and 2 days after consumption the symptoms appeared	*A. pegreffii*	Endoscopy	PCR (COII and EF1 α-1 nDNA region)	Colon	Sense of oppression in the right side of the abdomen	Removal by endoscopy/prompt recovery, no complications
	M	35	Tuscany, Livorno	Home-made raw marinated anchovies consumed 10 days prior to hospital admission	*A. pegreffii*	Laparatomy	RT-PCR hydrolysis probe system – based on mtDNA cox2	Colon	Abdominal pain, nausea and fever	Surgery/n.a.
Palma et al. [89]	F	52	Latium, Rome (H)	Raw anchovies consumed at a sushi restaurant 5 days before	*Anisakis* sp.	Endoscopy	Histology	Gastric	Epigastric pain and gastroesophageal reflux disease	Removal by endoscopy/n.a.

H: geographical location attributed on the basis of the hospital; n.a. = not available.
